# Deep learning generates custom-made logistic regression models for explaining how breast cancer subtypes are classified

**DOI:** 10.1371/journal.pone.0286072

**Published:** 2023-05-22

**Authors:** Takuma Shibahara, Chisa Wada, Yasuho Yamashita, Kazuhiro Fujita, Masamichi Sato, Junichi Kuwata, Atsushi Okamoto, Yoshimasa Ono

**Affiliations:** 1 Research and Development Group, Hitachi Limited, Tokyo, Japan; 2 Bioinformatics Group, Translational Research Department, Daiichi Sankyo RD Novare Coporation, Limited, Tokyo, Japan; 3 Translational Research Department, Daiichi Sankyo RD Novare Coporation, Limited, Tokyo, Japan; Bocconi University: Universita Bocconi, ITALY

## Abstract

Differentiating the intrinsic subtypes of breast cancer is crucial for deciding the best treatment strategy. Deep learning can predict the subtypes from genetic information more accurately than conventional statistical methods, but to date, deep learning has not been directly utilized to examine which genes are associated with which subtypes. To clarify the mechanisms embedded in the intrinsic subtypes, we developed an explainable deep learning model called a point-wise linear (PWL) model that generates a custom-made logistic regression for each patient. Logistic regression, which is familiar to both physicians and medical informatics researchers, allows us to analyze the importance of the feature variables, and the PWL model harnesses these practical abilities of logistic regression. In this study, we show that analyzing breast cancer subtypes is clinically beneficial for patients and one of the best ways to validate the capability of the PWL model. First, we trained the PWL model with RNA-seq data to predict PAM50 intrinsic subtypes and applied it to the 41/50 genes of PAM50 through the subtype prediction task. Second, we developed a deep enrichment analysis method to reveal the relationships between the PAM50 subtypes and the copy numbers of breast cancer. Our findings showed that the PWL model utilized genes relevant to the cell cycle-related pathways. These preliminary successes in breast cancer subtype analysis demonstrate the potential of our analysis strategy to clarify the mechanisms underlying breast cancer and improve overall clinical outcomes.

## Introduction

Seven decades after the birth of the learning machine [1], deep learning has evolved to the point that it can provide various predictive analyses. As deep learning spreads into more and more applications, including bioinformatics analysis, there is a growing need to explain the reasons for its predictions. Many methods that evaluate the importance of individual features have been devised to make deep learning models more explainable. These methods can be roughly classified into perturbation-based and saliency-based. In both types, the importance is determined by how much each feature contributes to the output. Perturbation-based methods calculate an importance score based on how the output behaves in relation to a perturbed input [2–4]. In saliency-based methods, the importance score depends on each feature’s saliency evaluated by the gradient of the output with respect to the input [5–8].

In the current study, we developed a point-wise linear model (PWL) for innately explainable deep learning in RNA-sequencing (RNA-seq) analysis. Conventional deep learning models compute new feature vectors with a linear combination that sufficiently expresses the objective model, while in contrast, the network of the proposed PWL model derives a weight function for each original feature vector as a function of the original feature vectors. Specifically, it generates a custom-made linear model (e.g., logistic regression) for each sample, and unlike a simple linear model, each linear model it generates includes the nonlinear interactions between the original features. Thus, the custom-made logistic regression models can solve the nonlinear classification tasks (e.g., the XOR problem introduced in [9]) that cannot be solved by conventional logistic regression. At the same time, the importance of each feature can be determined by its weight function within a specially designed logistic regression model. This property is particularly advantageous in the field of medicine, as medical researchers can benefit from the extensive knowledge and experience accumulated through the long use of logistic regression-based analysis. They can apply our proposed piecewise linear model in the same way as traditional logistic regression. Additionally, when the PWL model is utilized for deep learning that accurately predicts cancer subtypes, it can potentially access unknown and nonstandard knowledge related to gene expressions. Although the concept of factor analysis utilizing PWL was examined in [10], the technical details of PWL were not discussed. In this paper, we describe all the technical details of the PWL model and evaluate its technical validity through subtype analysis of breast cancer. Moreover, we have developed a novel deep enrichment analysis utilizing PWL to investigate the relationship between breast cancer subtypes and genes.

Breast cancer is the most frequently found cancer in women and is the type of cancer most often subjected to genetic analysis. Even so, it is a leading cause of cancer-related deaths in women. Analyzing breast cancer is clinically beneficial for patients and one of the best ways to validate the capability of the PWL model. Conventionally, breast cancer has been classified on the basis of the protein expression of the estrogen receptor (ER), progesterone receptor (PR), and epidermal growth factor receptor ErbB2/Her2, and expressions of these receptors have been used as clinicopathological variables for treatment decisions [11]. Since the early 2000s, high-throughput genomics technologies have demonstrated that breast cancer has five clinically relevant molecular subtypes defined by intrinsic gene expression patterns of the cancer [11–15]: Luminal A, Luminal B, Her2-enriched, basal-like, and normal breast-like cancer. While the subtypes do not perfectly reflect the clinical features, most breast cancers of the luminal subtypes are ER/PR-positive, most Her2-enriched ones have amplification of the Her2 gene, and most basal-like ones are triple negative (ER–/PR–/Her2–). In the original PAM50 study, the classification of the normal breast-like cancer subtype was trained with normal breast tissue [14]. Therefore, cancer samples classified to the normal-like subtype are often interpreted as low tumor content samples [14, 16].

There is a growing interest in utilizing machine learning models to predict prognosis and detect biomarkers for breast cancer patients using various data types, such as gene expression data and transcriptomic profiles [17–19]. We examine three methods in this work: two conventional machine learning methods and one deep learning method. The first method is the regularized Cox proportional hazards (RCPH) model, which uses gene expression data to identify prognostic biomarkers for breast cancer [17]. The RCPH model utilizes a regularization term to prevent overfitting. However, as a family of linear models, the RCPH model cannot handle nonlinear relationships between feature vectors. The second method is an ensemble model consisting of logistic regression, support vector machines (SVMs), and random forest, which is used to predict response to therapy using the multi-omic features of breast cancer patients [18]. This method facilitates the development of predictive biomarkers from multi-omic features. Ensemble learning effectively improves the prediction accuracy, but analyzing the rationale behind individual model predictions when integrated into a single outcome remains challenging. The third method utilizes DeepCC and is a deep learning-based cancer subtype classification method [19]. In two case tasks involving colorectal and breast cancer classification, DeepCC outperformed conventional machine learning models such as SVMs. DeepCC’s network architecture is a custom model derived from self-normalizing neural networks (SNNs) [20] to classify cancer subtypes accurately. Since DeepCC inherits the characteristics of SNNs, it does not directly output the feature importance in the prediction process. In summary, there are still challenges in balancing high prediction performance and explainability from machine learning and medical research perspectives. We designed PWL as a single model that combines high predictive performance with explainability to address these issues.

To evaluate the prediction performance of the PWL model, we prepared a classification task with RNA-seq values as the feature vectors and the five subtypes obtained from the PAM50 assay as the target variables. PAM50 was originally developed as a predictor of the five intrinsic subtypes from the expression pattern of 50 genes determined using a microarray [13]. If the important genes of the PWL model with RNA-seq values include the PAM50 genes, the PWL model will be semantically validated. While PAM50 subtyping is helpful for diagnosis and stratified treatment, it remains unclear which genes contribute to the mechanisms of action and/or mechanisms of resistance to treatment for each subtype. Copy number aberrations, i.e., deletion or amplification of large continuous segments of chromosomes, are a common type of somatic mutation in cancer and can be directly associated with the expression of genes and the development of cancer [21–24].

**Fig 1:**
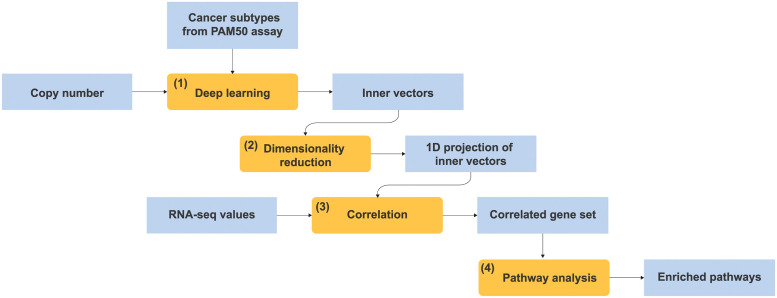
Processing pipeline of deep enrichment analysis method. Orange boxes are functions and processes. Blue boxes are input and output data. The deep learning model (orange box (1)) is a trained deep learning model (PWL) with copy number values as the feature vectors and subtypes as the target variables. The enrichment analysis process flow is as follows. (1) The deep learning model outputs the inner vectors of the hidden layer. (2) The dimensionality reduction function projects the inner vectors to 1-dimensional (1D) variables. (3) The correlation process extracts the highly correlated gene set between RNA-seq values and the projected 1D variables. (4) The pathway analysis enriches canonical pathways from the correlated gene set.

With advances in deep learning, highly accurate subtype prediction models can now be constructed from genomic data [19, 25]. Following their success, if the PWL model accurately predicts subtypes based on copy number, we can expect the underlying characteristics and mechanisms of cancer to be embedded in the PWL model. On the basis of this concept, we have newly developed a deep enrichment analysis method to understand the genes contributing to subtype classification and their mechanisms by considering the functions and pathways of genes. Fig 1 shows the processing pipeline of our deep enrichment analysis method. In the first step (Fig 1(1)), we prepared a deep learning model (PWL) with copy number values as the feature vectors and the subtypes as the target variables. In the following steps (Fig 1(2) and 1(3)), we calculated the correlation between the inner vector and the RNA-seq values to analyze the relationships between RNA-seq values and copy numbers. If the deep learning model can predict the subtypes of PAM50 derived from the mRNA expression level, valuable information to dictate the subtypes might be distilled in the inner vector of the deep learning model. In the final step (Fig 1(4)), we enriched canonical pathways from the highly correlated genes between RNA-seq values and copy numbers.

A paper overview (and guide for readers) is provided in S1 Fig., where the parts of the Materials and methods and Results sections dealing with the breast cancer subtype analysis are indicated in orange. Readers who want to grasp essential information rapidly can take a quick look at the sections and subsections indicated by check marks.

## Materials and methods

### Point-wise linear models

**Fig 2:**
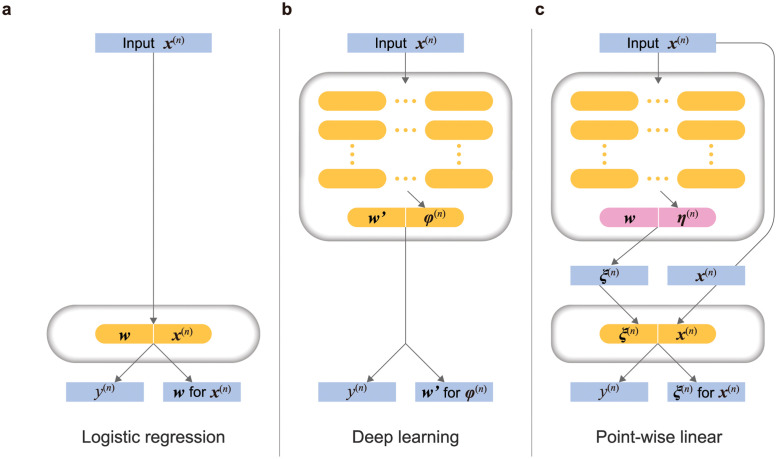
Comparison of network architectures. (a) shows a logistic regression model. ***x***^(*n*)^ and *y*^(*n*)^ indicate a feature vector and a target value ((*n*) is sample index), respectively. ***w*** is a vector of learning parameters for ***x***^(*n*)^. (b) shows a fully connected neural network. ***φ***^(*n*)^ and ***w***^′^ indicate an inner vector and learning parameters, respectively. (c) shows a PWL model. The upper block in (c) is a meta-machine generating a learning parameter ***ξ***(***x***^(*n*)^). The lower block in (c) is a logistic regression model for each feature vector ***x***^(*n*)^.

**Fig 3:**
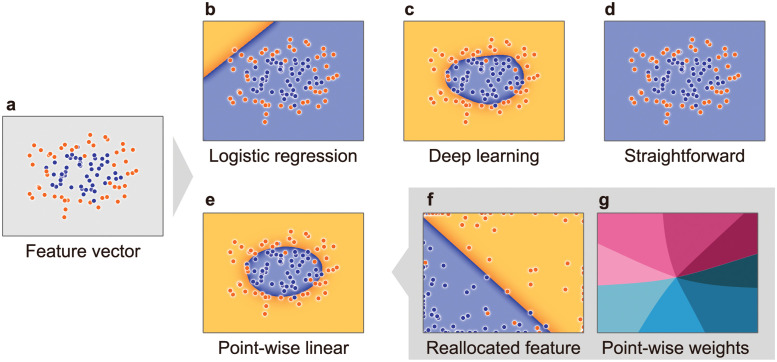
Comparison of learning ability and explainability. (a) is a large circle (orange dots) that contains a smaller circle (blue dots) obtained by *sklearn.datasets.make_circle*. (b) and (c) are the boundaries classified by the logistic regression model and self-normalizing network (SNN) model, respectively. (d) and (e) are the boundaries classified by the PWL model in a straightforward manner (Eq (3)) and by using the reallocation function (Eq (4)), respectively. (f) is the boundary classified by the reallocated feature vectors ***ρ***. (g) is the arctangent of the angle between the horizontal and vertical elements of the weight vector ***ξ***^(*n*)^. The weight vector smoothly changes for each data sample.

To investigate the nonlinear prediction ability and explainability of the PWL model, we trained a logistic regression model and a SNNs model as a state-of-the-art (SOTA) deep learning [20] using a simple dataset (a large circle containing a smaller circle generated by *sklearn*.*datasets*.*make_circle* [26]). Fig 2 shows three architectures of the machine learning models: (a) logistic regression, (b) deep learning, and (c) PWL. Let $x^{\left(n\right)}\in R^{D}$ represent a feature vector with *N* denoting the sample size and R indicating the real number set. First, we define a logistic regression model (Fig 2(a)) as

$$\begin{array}{c} y^{\left(n\right)}=\sigma \left(w\cdot x^{\left(n\right)}\right), \end{array}$$ (1)

where $w\in R^{D}$ is a weight vector for ***x***^(*n*)^, *σ* is a sigmoid function, and ⋅ is the inner product. *y*^(*n*)^ is a probability value such as one expressing the likelihood of tumor tissues or normal tissues. The weight vector ***w*** is bound to the feature vector ***x***^(*n*)^. We can determine the importance of each feature variable by analyzing the magnitude of the elements in ***w***. However, as shown in Fig 3, since the circle in a circle is not a linearly separable problem, the logistic regression model cannot classify the two circles.

Next, we define a standard deep learning model like that shown in Fig 2(b). A new feature vector $\varphi \left(x^{\left(n\right)}\right)\in R^{D^{\prime }}$ is nonlinearly generated from the original feature vector ***x***^(*n*)^ through the *L*-layer deep neural network. Typically, *L* is set from several layers to tens of layers. Note that the notation ***v***(***u***) for arbitrary vectors ***v*** and ***u*** indicates that every element of ***v*** is a function of the elements of ***u***. A deep-learning-based nonlinear classification function predicts the probability *y*^(*n*)^ (Fig 2(b)) as follows:

$$\begin{array}{cc} y^{\left(n\right)} & =\sigma (w^{\prime }\cdot \varphi \left(x^{\left(n\right)}\right)), \end{array}$$ (2)

where $w^{\prime }\in R^{D^{\prime }}$ is a universal weight vector for ***φ***. The magnitude of each ***w***^′^ element represents the contribution of the corresponding element of ***φ*** to the prediction, as shown in Fig 2(b). The SNNs correctly classified the blue and orange dots, as shown in Fig 3(c). However, we cannot “explain” the machine’s prediction by ***w***^′^ because it is not possible to understand the meanings of ***φ*** that the machine uses to make its predictions.

In order to make a deep NN explainable, we investigated a meta-learning approach to generate a logistic regression model defined (Fig 2(c)) as

$$\begin{array}{cc} y^{\left(n\right)} & =\sigma (\xi \left(x^{\left(n\right)}\right)\cdot x^{\left(n\right)}), \end{array}$$ (3)

where each element of $\xi \in R^{D}$ is a function of ***x***^(*n*)^ that the NN determines. ***ξ*** behaves as the weight vector for the original feature vector ***x***^(*n*)^. The magnitude of each element of ***ξ*** describes the importance of the corresponding feature variable. We should point out here that this weight vector is tailored to each sample because ***ξ*** depends on ***x***^(*n*)^. We call Eq (3) a PWL model given by a straightforward method over the sample index (*n*). The architecture of the PWL model consists of the two blocks shown in Fig 2(c). Also, we refer to ***ξ*** as the point-wise weight. The upper block is a meta-learning machine that generates logistic regression models, and the lower block shows the logistic regression models for the inference task. However, the tailored weight vector ***ξ*** can easily lead to poor generalization (i.e., over or underfitting). In this case, the PWL model (Eq (3)) tries to learn the labels of all samples because it generates a weight vector optimized for each sample, which leads to the underfitting shown in Fig 3(c).

We came up with a new equation that constructs a point-wise weight ***ξ*** without losing generalization ability, as follows:

$$\begin{array}{c} \xi \left(x^{\left(n\right)}\right)\equiv w⊙\eta \left(x^{\left(n\right)}\right), \end{array}$$ (4)

where the reallocation vector $\eta \in R^{D}$ is nonlinearly generated from the original feature vector ***x***^(*n*)^ through the *L*-layer NN (*L* ≥ 2). $w\in R^{D}$ is a universal weight vector that is independent of ***x***^(*n*)^, and ⊙ is the Hadamard product. The condition *L* ≥ 2 is given to ensure that the neural network satisfies the universal theorem of NNs in [27] and retains the ability to solve nonlinear problems in the PWL model. In our experiments using RNA-seq and the copy number features, the average number of internal layers was optimized as *L* = 17 and 19, respectively. In contrast to the model defined straightforwardly by Eq (3), the model defined by Eq (4) accurately predicts the classification boundary, as shown in Fig 3(e). The weight vectors ***ξ***^(*n*)^ in Eq (4) smoothly change for each data sample (Fig 3(g)). The reallocation-based PWL model thus enables generalization. Additionally, we call ***ρ***(***x***^(*n*)^) ≡ ***η***(***x***^(*n*)^) ⊙ ***x***^(*n*)^ a reallocated feature vector in $R^{d}$. NNs have the versatile ability to map a linear feature space to a nonlinear feature space. By utilizing this ability, ***η***^(*n*)^ reallocates the feature vector ***x***^(*n*)^ into the new vector ***ρ*** that is linearly separable by a single hyperplane drawn by ***w***.

The family of linear regression models is given by the inner product of the weight ***w*** and the feature vector ***x***, as shown in Eq (3). Thus, PWL can handle logistic regression, linear support vector machines, elastic-net, and Cox regression. Additionally, functions such as CNNs and RNNs that satisfy the universal approximation theorem can be used as ***η*** functions accordingly. In the following experiments, we used deep unified networks (DUNs) [28] as ***η***. The mechanisms underlying Eq (4) and the DUNs are discussed in S1 Appendix. PWL was implemented as a Python library, and the source code and instructions for running a PWL model locally are available on GitHub [29]

### Datasets

To validate the PWL model, we used the breast cancer TCGA [30] dataset retrieved by the UCSC public Xena hub [31] for the gene expression RNA-seq dataset (dataset ID: TCGA.BRCA.sampleMap/HiSeqV2_PANCAN), the copy number alteration (gene-level) dataset (dataset ID: TCGA.BRCA.sampleMap/Gistic2_CopyNumber _Gistic2_all_thresholded.by_genes), and the phenotype dataset (dataset ID: TCGA.BRCA.sampleMap/BRCA_clinicalMatrix). RNA-seq values were calculated by UCSC Xena as follows. *Log*_2_(*x* + 1) values were mean-normalized per-gene across all TCGA samples (*x* is RSEM normalized count [32]). In the copy number dataset of UCSC Xena, GISTIC2 values were discretized to −2, −1, 0, 1, 2 by Broad Firehose. We used subtypes pre-calculated by PAM50 (Luminal A, Luminal B, basal-like, Her2-enriched, and normal-like [12]) in the Xena dataset as the target variables of the prediction model [15]. Table 1 lists the number of samples for each subtype. The number *D* (feature dimension) of mRNAs types was 17,837, as we adopted gene symbols that overlap with both the RNA-seq data and the copy number alteration data.

**Table 1:** Number of samples for each breast cancer subtype (Total = 810).

	Normal-like	Luminal A	Luminal B	Basal-like	Her2-enriched
Number of samples	22	406	185	131	66

### Feature importance calculation method

We utilized the PWL model (Eq (3)) to calculate the importance of each feature variable, i.e., how much each feature contributes to the model’s prediction. The point-wise weight vector ***ξ*** depends on ***x***^(*n*)^ and consequently describes each sample’s own feature importance. Therefore, we came up with a method to derive the feature importance for a sample group so as to reveal both the group and the macroscopic property contained in the point-wise weight vector of the group’s samples. This concept of the feature importance was also used in [10].

First, we calculated the feature importance for each sample from the weight vector ***ξ***^(*n*)^ in Eq (3). Inspired by the Shapley value [33], we introduced a sample-wise importance score for the *k*-th feature *x*_*k*_ of a sample with index (*n*) as

$$\begin{array}{cc} s_{k}^{\left(n\right)} & \equiv \xi_{k}^{\left(n\right)}x_{k}^{\left(n\right)}-\frac{1}{|U_{\left(n\right)}|}\sum_{i\in U_{\left(n\right)}}[\xi_{k}^{\left(i\right)}x_{k}^{\left(i\right)}], \end{array}$$ (5)

where *U*_(*n*)_ is the set of samples whose weights are close to those of sample (*n*). This sample-wise importance score expresses the contribution of a sample (*n*) to raising the output probability *y*^(*n*)^ compared with the average contribution among a sample group whose members obey similar linear models. In this study, we defined *U*_(*n*)_ as follows: a sample (*i*) is in the set *U*_(*n*)_ if |***ξ***^(*i*)^ − ***ξ***^(*n*)^|/|***ξ***^(*n*)^| is smaller than $4|\sigma |/|\bar{\xi }|$, where ***σ*** and $\bar{\xi }$ are vectors whose elements are given as the standard deviation and the mean, respectively, of the corresponding element of ***ξ***.

Next, we defined group-wise importance scores for each group (e.g., subtype Her2 samples) by using the sample-wise importance score. We implemented voting among the group, where each sample votes on its top 10% features as to which sample-wise importance scores are the highest, i.e., the features that significantly raise each sample’s output probability. We defined a group-wise importance score *v* for each feature as the rate of samples who vote for the feature in the above voting.

Finally, we introduced a relative score to extract the features that characterize a subtype. We divided the samples into samples of a target subtype and others and then evaluated the group-wise importance score for each group. We refer to the group-wise importance score for the target subtype samples group as *v*_target_ and the one for the others as *v*_others_. *v*_target_ is not necessarily appropriate for extracting the features that characterize the target subtype because even when *v*_target_ is high for a feature, if *v*_others_ is also high, the feature might be important for all the subtype samples, not just for the target ones. Therefore, we defined a relative score *v*_rel_ so as to compute the feature importance for the subtype samples relative to the one for the others, as

$$\begin{array}{cc} v_{\text{rel}} & \equiv {\left(v_{\text{target}}\right)}^{2}-{\left(v_{\text{others}}\right)}^{2}. \end{array}$$ (6)

Since the range of both *v*_target_ and *v*_others_ is [0, 1], the range of *v*_rel_ becomes [−1, 1]. S2 Fig. shows the distribution of *v*_rel_ in the *v*_others_-*v*_target_ space. If a relative score is large, we can expect that both the summation and difference of the group-wise importance scores *v*_target_ and *v*_others_ will be large as well. In other words, features with large relative scores are important to a certain degree for all the samples, and simultaneously they are much more important for the target subtype samples than for the others. We considered the features with high relative scores for each subtype to be the important features that characterize the corresponding subtype.

### Deep enrichment analysis method

In addition to the importance scores, the PWL model implements a deep learning-based enrichment analysis method to extract the canonical pathway in the nature of the breast subtypes, as summarized in Fig 1. Prior to the enrichment analysis, we trained the deep learning model (indicated by the orange box (1)) with copy number values as the feature vectors and the subtypes as the target variables. Step (1) of the pipeline utilizes the inner vectors of the hidden layer. The outputs of the NN (deep learning) inner layers give us some hints as to what criteria the NNs use to classify the subtypes of breast cancer from the feature vector. The output of the final inner layer (a new feature vector ***φ***) can be linearly separated by a single hyperplane spanned by ***w***^′^, as shown in Eq (2). When the prediction accuracy is high, the output of the final inner layer provides a well-summarized representation of the feature vector ***x***1 for the classification task. We utilized the reallocation vector ***η*** as the inner vectors, as shown in Fig 1 (the output of (1)).

To determine the subtype classification criteria, the second step of the pipeline (Fig 1(2)) utilizes a manifold learning technique called UMAP for the dimensionality reduction [34]. The UMAP then compresses the reallocation vectors ***η*** into a one-dimensional (1D) vector. The third step (Fig 1(3)) then calculates the Spearman’s correlation coefficient for the relationship between the 1D vector (the projection of ***η***) and RNA-seq values, after which we select the top 250 genes for which the correlation coefficient was positive and the top 250 genes for which the correlation coefficient was negative. The final step of the pipeline (Fig 1(4)) analyzes these genes by using Ingenuity Pathway Analysis (IPA, QIAGEN, [35]) to interpret the canonical pathways.

### Evaluation methodology

We compared the performance of the proposed PWL with that of three state-of-the-art models and one widely used model for subtype prediction. The first was SNNs, a SOTA deep learning model developed for general tasks [20]. The second was DeepCC, a deep learning-based cancer subtype classification model [19]. Note that we used the deep learning model of DeepCC (i.e., without the functional spectra) so as to compare the pure performance of the deep learning models. The third was DeepCSD, which utilizes a deep learning model with a regularization mechanism to prevent overfitting [25]. The fourth was logistic regression, a standard model commonly used in bioinformatics. Recall that the PWL method was developed as a set of custom logistic regression models to solve nonlinear classification tasks. The subtype prediction models were built using logistic regression with regularization (implemented by scikit-learn v0.22, Python 3.7.6), SNNs (implemented by PyTorch 1.5, Python 3.7. 6), DeepCC (implemented by PyTorch 1.11.0, Python 3.9.10) [19], DeepCSD (implemented by PyTorch 1.11.0, Python 3.9.10) [25], and PWL (implemented by PyTorch 1.5, Python 3.7.6).

If the hyperparameters of a prediction model (e.g., the number of layers) are optimized by using all samples, we may overlook the hyperparameter overfitting. To address this issue, we carried out the prediction model evaluation by a *K*-fold double cross-validation (DCV) [36]. The *K*-fold DCV can measure the prediction performance of the entire learning process including its hyperparameter optimization. The procedure of *K*-fold DCV has internal (training) and outer (test) loops. In this study, each internal loop searches for the best hyperparameter set (i.e., best combinations of the hyperparameters) of the prediction model *L* times by using a tree-structured Parzen estimator [37, 38], where a single nested loop in the inner loops uses *M*-fold CV to evaluate the prediction performance of the prediction model with a hyperparameter set. Each inner loop trains the prediction models with different hyperparameter sets *L* × *M* times. Then, each outer loop tests the prediction model with the best hyperparameter set obtained by its internal loop. The hyperparameter optimization process is thus completely separated from the test data. In our experiment, we set M = K = 10 and L = 100, and trained the prediction model by 10,000 (*K* × *L* × *M*) times.

In each iteration of the 10-fold DCV and its internal 10-fold CV, the mean area under the curve (AUC) was calculated as

$$\begin{array}{c} \text{Mean}\;\text{AUC}=\frac{1}{K}\sum_{k=1}^{K}\frac{1}{C}\sum_{t\in subtypes}\text{AUC}\left(k,t\right), \end{array}$$ (7)

where *subtypes* is a set of subtype categories (*C* = 5: Normal, Luminal A, Luminal B, Basal and Her2), and AUC(*k*,*t*) is the *k*-th and subtype *t*’s AUC. In addition, we used Tukey’s honest significance test (implemented by Python 3.9.10 and Statsmodels 0.13.2) for the multiple comparison to clarify the DCV results of the machine learning models. We set the family-wise error rate (FWER) threshold to less than 0.05 for the Tukey’s honest significance test.

According to the IPA’s help and support pages, the p-value is calculated using the right-tailed Fisher Exact Test. The p-value for a pathway is thus calculated by considering:

1. the number of genes that participate in that pathway, and
2. the total number of genes in the QIAGEN Knowledge Base that are known to be associated with that pathway.

## Results

### Prediction performance

Prior to the breast cancer subtype analysis, we evaluated five subtype prediction models: logistic regression with regularization, SNNs, DeepCC, DeepCSD, and the proposed PWL. Table 2 lists the AUC values of the 10-fold DCV for the RNA-seq and copy number features. All data from the 10-fold DCV and multiple comparison tests were stored in S1 File. The average number of internal layers in the PWL models was optimized to *L* = 17 and 19 for the RNA-seq and copy number features, respectively. Each prediction model’s hyperparameter search ranges and values are stored in S2 File. The values in the ‘All’ column in Table 2 were calculated using Eq (7). The AUC value of each subtype was then averaged over the 10-fold DCV. As we can see, all prediction models (excluding DeepCSD) with the RNA-seq features achieved an AUC greater than 0.90. The PWL model had statistical significance compared to the SNNs and DeepCSD models (FWER < 0.05), but had no statistical significance compared to the logistic regression and DeepCC models. The PWL model with the copy number features marked the best values (training: 0.859, test: 0.862) shown in ‘All’. The AUC values of the other deep learning models were below 0.8. The logistic regression and the PWL model had no statistical significance, but both had statistical significance compared to the SNNs, DeepCC, and DeepCSD models (FWER < 0.05).

**Table 2:** Mean AUC values of 10-fold DCV results.

Features	Models	Training All	Test					
			All	Normal	Luminal A	Luminal B	Basal	Her2
RNA-seq	Logistic	**0.983**	**0.985**	**0.984**	**0.977**	**0.972**	**0.999**	**0.993**
	SNNs	0.933	0.930	0.843	0.948	0.930	0.990	0.940
	DeepCC	0.962	0.961	0.903	0.955	0.941	0.999	0.989
	DeepCSD	0.910	0.898	0.700	0.915	0.848	0.998	0.806
	PWL	0.980	0.975	0.956	0.966	0.964	**0.999**	0.992
Copy number	Logistic	0.851	0.853	**0.883**	0.805	0.746	0.985	0.845
	SNNs	0.772	0.745	0.644	0.752	0.615	0.972	0.742
	DeepCC	0.797	0.791	0.672	0.784	0.681	0.979	0.809
	DeepCSD	0.725	0.718	0.581	0.767	0.477	0.939	0.697
	PWL	**0.859**	**0.862**	0.879	**0.817**	**0.779**	**0.986**	**0.848**

Logistic: Logistic regression

Normal: Normal-like

Basal: Basal-like

Her2: Her2-enriched

### Importance analysis

We further investigated the PWL model by comparing the overlap between the important genes and the PAM50 genes. Then, the top 500 genes in terms of the relative score (stored in S3 File) calculated by the importance analysis (see Feature importance calculation in the Methods section) were selected as highly contributing feature variables to predict subtypes. We then checked how many PAM50 genes were included (S5 Fig). Table 3 summarizes the overlap rate between the Top 500 and the PAM50 genes. As expected, when trained with RNA-seq, both the logistic regression and PWL models contained 44 and 41 of PAM50 genes, respectively, but when training on copy number data, the overlaps of PAM50 genes were low: 14 and 15 out of 50 genes, respectively. Note that we analyzed the deep learning models using UMAP, as discussed in the following ‘Enrichment analysis’ section.

**Table 3:** Number of overlaps between PAM50 genes and top 500 important genes in each model.

Features	Models	All	Normal	Luminal A	Luminal B	Basal	Her2
RNA-seq	Logistic	44	10	27	22	15	13
	PWL	41	7	22	22	10	13
Copy number	Logistic	14	1	3	4	3	8
	PWL	15	5	3	1	1	7

Logistic: Logistic regression

Normal: Normal-like

Basal: Basal-like

Her2: Her2-enriched

**Fig 4:**
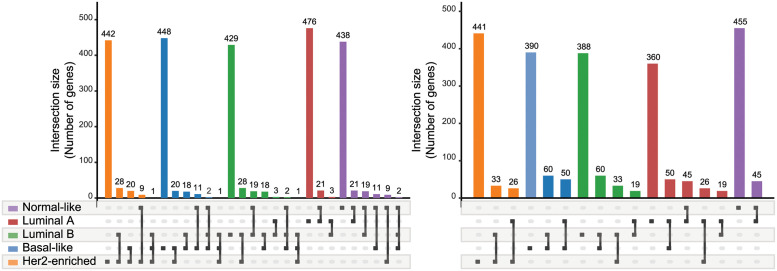
Intersections of top 500 gene sets calculated by importance analysis of the copy number. (a) PWL model. (b) Logistic regression model.

To examine which aspects of these contributing feature genes differed, in each model trained with copy number data, we compared those genes across the subtypes in Fig 4 (S3 Fig. shows the results of the RNA-seq). We found that the majority of the contributing feature genes were specific in certain subtypes because they were not selected in other subtypes. In addition, a comparison between modeling methods showed that the PWL model tended to have a higher proportion of the specific genes than the logistic regression model for all tumor subtypes. Duplication of the specific genes between the two models showed not much commonality with 206 genes even in the most overlapping Her2 subtype, as summarized in Table 4 (S1 Table summarizes the results of the RNA-seq). The Her2 subtype is a class in which the amplification of the HER2 gene is enriched, and many genes in the vicinity of the HER2 (also known as ERBB2 and is located on chromosome 17) gene were commonly contained (please see the ideogram of chromosome 17 as shown in S6 Fig). S2 Table summarizes the chromosomal location for the common genes as the specific features of the Her2-enriched class in the logistic regression and PWL models. Since both models were generated from copy number data, it is reasonable that they had a relatively high degree of commonality.

**Table 4:** Commonalities of specific feature genes for copy number.

		PWL				
		Normal-like	Luminal A	Luminal B	Basal-like	Her2-enriched
Logistic	Normal-like	37	21	0	0	0
	Luminal A	10	43	39	3	0
	Luminal B	4	0	117	7	4
	Basal-like	2	60	0	82	3
	Her2-enriched	6	3	3	3	206

Logistic: Logistic regression

### Enrichment analysis

The previous subsection compared the PWL and logistic regression models by evaluating the overlap rates of the PAM50 genes. As Table 3 shows, the PWL model with the copy number features used genes other than the PAM50 genes for subtype classification. In this subsection, we performed enrichment analysis to determine which pathways were used by the PWL model with the copy number features to classify the subtypes. We then compared the pathways enriched by the PWL model and the other deep learning models (SNNs, DeepCC, and DeepCSD).

**Fig 5:**
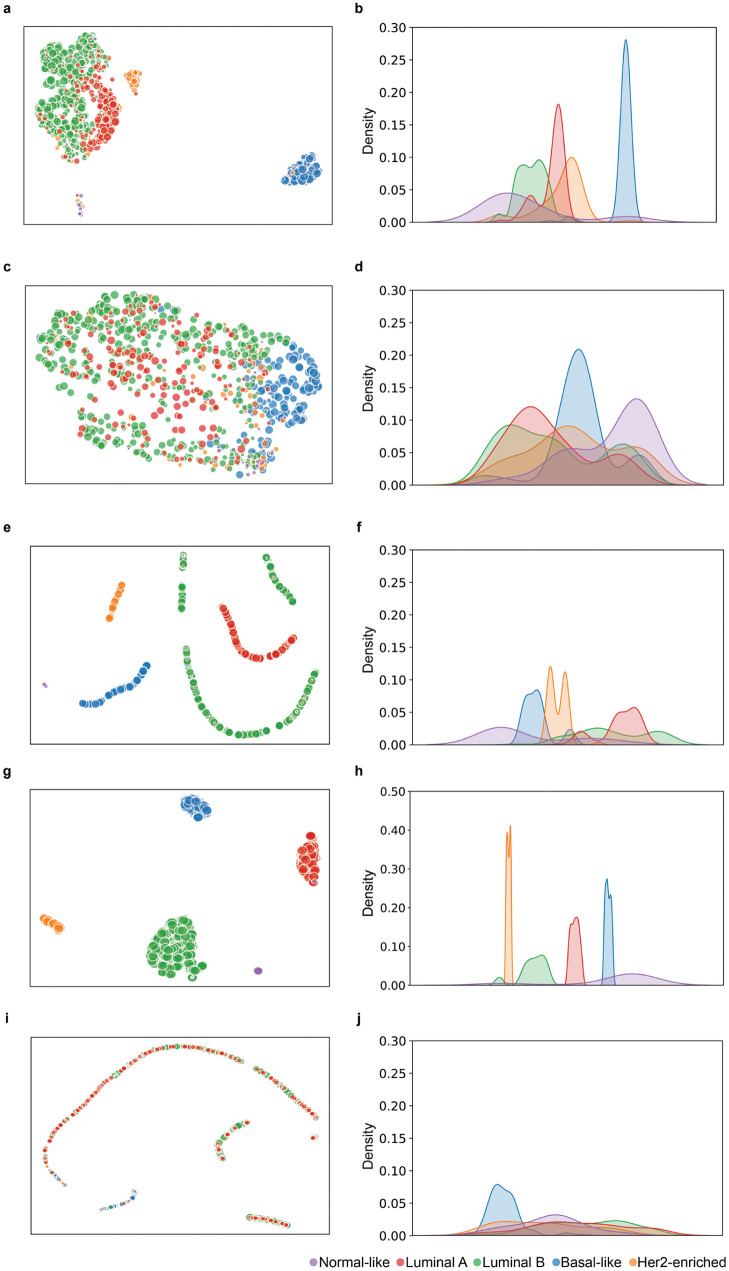
Embeddings of deep learning models with copy number features projected by UMAP. The left and right columns are 1D and 2D projected embeddings, respectively. The first to fifth rows show embeddings of RV ***η***, RF ***ρ***, SNNs, DeepCC, and DeepCSD, respectively.

Fig 5 shows the 1D and 2D embeddings of the reallocation vector (RV ***η***) and the reallocated feature (RF ***ρ***, and the final inner vector of the deep learning models. (***φ*** in Eq (2)) for the copy number features (S4 Fig shows the results of the RNA-seq). The RV vector reflected the clinical features (ER–/PR–/Her2–). As shown in Fig 5(a), Luminal A and B were stuck together, but each peak of their 1D embeddings stood in a distinct position. Her2-enriched samples were close to the clusters of the Luminal A and B samples. The 2D embedding of RV ***η*** (Fig 5(a)) shows that the basal-like samples stayed away from other subtypes. Normal-like samples took a position under the Luminal and Her2-enriched samples. In the case of RF***ρ*** embeddings, all subtypes formed a single cluster, as shown in Fig 5(c). The embeddings of the SNNs and DeepCC models were separated for each subtype, as shown in Fig 5(e) and 5(g). The embeddings of the DeepCSD model formed strings that mix the different subtypes samples, as shown in Fig 5(i).

We obtained each of the top 500 genes correlated with the mRNA expression values and then examined their functionally enriched pathways using IPA. These subtypes were originally grouped by PAM50 on the basis of the mRNA expression level. For this reason, we investigated which kind of mRNA expression levels of the genes were associated with the 1D embeddings of RV ***η***, RF ***ρ***, and the inner vector of the deep learning models to figure out the differences between the four vectors focusing on models from only copy number data. We have summarized the results of PWL, SNNs, and DeepCC in order of prediction accuracy in Table 5 (the full list is available in S4 File). The order of the canonical pathway is sorted by the −*log*_10_(*p*-value) of the PWL RV ***η*** embeddings, as the prediction accuracy of the PWL model was statistically significant compared to the other models (FWER <0.05), and the values of the PWL RV ***η*** embeddings were higher than those of the RF ***ρ***.

**Table 5:** Enriched pathways of the top 500 genes’ mRNA expression level associated with the 1D embeddings of RV *η*, RF *ρ*, and the inner vectors of SNNs and DeepCC in UMAP.

Canonical pathways in IPA	−*log*_10_(*p*-value)			
	RV *η*	RF *ρ*	SNNs	DeepCC
Kinetochore metaphase signaling pathway	20.59	2.04	5.39	21.80
Cell cycle: G2/M DNA damage checkpoint regulation	10.62	0.00	0.00	7.12
Estrogen-mediated S-phase entry	10.21	0.37	2.69	10.21
Mitotic roles of Polo-like kinase	10.00	0.00	0.38	12.31
Cell cycle control of chromosomal replication	9.95	0.00	0.00	7.69
Role of CHK proteins in cell cycle checkpoint control	8.57	0.27	0.98	7.59
Cyclins and cell cycle regulation	8.66	0.28	1.00	7.59
Role of BRCA1 in DNA damage response	6.96	1.05	0.00	9.96
Ribonucleotide reductase signaling pathway	6.90	0.82	1.55	4.81
Cell cycle: G1/S checkpoint regulation	6.78	0.00	1.84	4.91

The top 9/10 pathways were the same between the PWL RV ***η*** and DeepCC embeddings. Those genes were overlapped with cell cycle-related pathways such as “Kinetochore metaphase signaling pathway”, “G2/M DNA damage checkpoint regulation”, “Estrogen-mediated S-phase entry”, “Mitotic roles of Polo-like kinase”, “Ribonucleotide reductase signaling pathway”, and “Cell cycle control of chromosomal replication”. In contrast, the ones derived from RF ***ρ*** showed little significant enrichment. The gene from the SNNs embeddings that enriched pathways suggested the occurrence of a cell cycle during growth and development; however, its statistical significance was low. The cell cycle is an essential function of cell proliferation and affects the characteristics and malignancy of cancer [39]. The PWL, SNNs, and DeepCC models recognized these pathways in the subtype classification, and both the PWL and DeepCC models actually represented multiple related pathways. We performed the same analysis for the PWL model generated from RNA-seq data only (the enriched pathways were stored in S4 File) and found that RV ***η*** genes from RNA-seq/copy number data more significantly enriched the cell cycle pathway.

## Discussion

For the RNA-seq features, the logistic regression model was a better predictor of the PAM50 subtypes than the deep learning models, as summarized in Table 2. This result is reasonable because the subtypes as the target variables were detected from the RNA-seq expression pattern analysis through the PAM50 assay. The significant relationships between the RNA-seq features and the subtypes were relatively simple and suitable for the logistic regression model. On the other hand, the feature vector size and the configuration of the large dimensional feature size (*D* = 17, 837) and small sample size (*n* = 810) decreased the generalization ability of the deep learning models (training: 0.980, test: 0.975). Here, a more meaningful result than the prediction accuracy was that the deep learning model used 41 genes of PAM50 to classify the subtypes, as summarized in Table 3. The reasoning was that the PWL model with the RNA-seq features uses the PAM50 genes to predict the subtypes derived from the PAM50 assay. These results demonstrated that the PWL model was a semantically valid model and encouraged us to apply the PWL model to reveal the relationships between the copy number features and the subtypes.

PAM50 subtype prediction from the copy number features is not a trivial task compared to prediction from the RNA-seq. The AUC values of the PWL model were better than those of the logistic regression and deep learning models throughout the 10-fold DCV. All three deep learning models (excluding the PWL model) experienced overfitting, as indicated by the higher AUC values in the training results and the lower AUC values in the test results (Table 2). One possible reason for this overfitting is that the progression of learning is confined to the upper-layer parameters: i.e., there is a lack of advanced learning in the lower layers. Klambauer et al. showed that SNNs could use 32 layers in the conventional machine learning dataset [20]. In our task, the number of the SNN model’s inner layers (average 13, minimum 10, and maximum 16, as summarized in S2 File) was lower than that of the PWL model (average 19, minimum 12, and maximum 24, as summarized in S2 File). We thus optimized the number of both models’ inner layers from 10 to 25. As shown in Fig 2, the PWL model contains a deep learning block to generate the custom-made logistic regression model, so its model likely causes overfitting when the number of layers is increased. In contrast, the DeepCC and DeepCSD models were developed using thin-layer networks (DeepCC: five inner layers and one output layer, DeepCSD: two inner layers and one output layer). The prediction performance of both models suggested that thin layers could not effectively represent the relationships between copy number and subtypes, indicating that they faced challenges in handling the inner layers regardless of whether the number of layers was thin or thick. For the PWL model, we used a unified architecture (see S1 Appendix and [28]) as a deep learning architecture, which is characterized by the interconnection of each network layer with neurons arranged in a mesh-like form. This unified architecture has horizontally shallow and vertically deep layers to prevent gradient vanishing and explosion. No matter how many layers are stacked vertically, there are only two horizontal layers from the data unit neurons to the output neurons.

The logistic regression model and the PWL model had almost equivalent prediction performances for the copy number data (FWER ≥0.05 stored in S1 File). On the other hand, Table 4 suggests that the genes important for predicting the breast cancer subtypes were different in the two models: namely, the PWL model tended to select specific genes for each subtype, as shown in Fig 4. This difference is considered to stem from the models’ ability to treat the nonlinear relationships between feature and target variables. The logistic regression expresses the target variables only as linear combinations of the feature variables, while the PWL can express the target variables nonlinearly, as in Eqs (3) and (4). In addition, these two equations can be modified as ***w*** ⋅ (***η***(***x***) ⊙ ***x***). The RV ***η*** corrects the feature variables ***x*** so that the universal weight ***w*** can linearly separate the feature vector ***x***. The differences of important genes between the PWL and logistic regression methods stem from the feature variables’ correction mechanism of the PWL method. This mechanism might also help the PWL model express the target variables without using some of the features not specific to the subtype. In contrast, the logistic regression model has to use nonspecific features to express the target variables without the corrections. We expect the PWL model’s correction mechanism to be vital in tasks that demand high nonlinearity and consequently result in low AUC values in logistic regression. One of the tasks in this study was to predict subtypes Luminal A and B (see Table 2). Consistently, the difference of the number of specific genes in the two models was large in Luminal A and B, and the specific genes for Luminal A and B in the PWL model had much in common with the specific genes of other subtypes in the logistic regression model, as shown in Table 4.

As discussed above, the results in Fig 4, Tables 2 and 4) are consistent with the interpretation of the PWL model from the viewpoint of the correction to the features. This consistency suggests that our scoring method (described in ‘Feature importance calculation method’ in the Methods section) works properly. Our scoring method was designed to extract the features that contribute to predicting a subtype, especially among the corresponding subtype samples, rather than features not found in the other samples. This scoring method helps us clarify which features contribute to the prediction result with the aid of corrections by RV ***η***, but we cannot examine which features contribute to the corrections ***η***. Therefore, the relative score is not a perfect measure to investigate the mechanism of the classification of breast cancer subtypes.

For further investigation, we analyzed our predictive model’s internal state in detail by using the deep enrichment analysis method, as shown in Fig 1. The results suggest that the gene sets’ biological implications contribute to the classification. From the comparison of RV ***η***, RF ***ρ*** as the PWL model’s inner vectors to be analyzed, we found that RV ***η*** was better suited to elicit candidate hypotheses. As shown in Fig 1(2) through 1(4), we utilized UMAP to analyze the internal state in detail and reduce the dimensionality and then combined it with mRNA expression levels. The results of importance analysis (S3 File, S2 Table, and see above) did not indicate the presence of specific genes (except for Her2) that determine subtypes in breast cancer. However, the results of enrichment analysis derived the biological implication that ***η*** was involved in the cell cycle. The fact that genes involved in the cell cycle affect subtypes is well-known [40], and such genes have been reported as promising drug targets [39, 41]. The regulatory network of cell cycle control is diverse and complex, featuring multiple checkpoints in response to DNA damage, growth signaling, replication stress, etc. [41]. Subtype-specific differences in the genetic variation might occur in the complex network of cell cycle control, which may be reflected in the UMAP 1D projection. These findings indicate that the internal state of our model was worth analyzing.

Note that the UMAP embedding of RV ***η***, rather than the ones of RF ***ρ*** and ***φ***, contains richer information related to interpretable pathways. While RF ***ρ*** is created as new features with which the problem is linearly separable in the PWL model (as well as ***φ*** in the SNNs and DeepCC models), RV ***η*** is considered as the corrections to the features by which the features are transformed into RF ***ρ***, and is unique to the PWL model. This finding suggests that it is essential to analyze the corrections to the features for our task in order to investigate the breast cancer subtypes classification mechanism. Regarding further analysis or hypotheses, the PWL model’s ***η*** is preferred, as it presented multiple related pathways in this report. Considering this case as an example, we feel confident that biologists and informaticians can apply our analysis with RV ***η*** to other tasks, such as exploring new drug target molecules or investigating mechanisms.

The limitation of this study is the retrospective analysis of the breast cancer data. All results obtained from the prediction models should be evaluated by clinical practice as a prospective study. Even so, the results of this retrospective study demonstrate the potential of our technique to reveal unknown and nonstandard knowledge of breast cancer.

## Conclusion

This study has established the PWL model as an innately explainable deep learning model that can analyze the biological mechanisms of breast cancer subtypes. The PWL model generates a custom-made logistic regression model, which allows us to analyze which genes are important for each subtype of an individual patient. We presented a new scoring method for selecting genes for the subtype classification, and also demonstrated that the deep enrichment analysis method with the PWL model can extract the genes relevant to cell cycle-related pathways. Our PWL model utilized as explainable deep learning can reveal the mechanisms underlying cancers and thereby contribute to improving overall clinical outcomes.

## Supporting information

S1 Fig Paper overview. The orange background sections show the methods and results of the breast cancer subtypes analysis. Check-marked sections provide essential information about our paper.
(EPS)

S2 Fig Heat map visualization of distribution of relative score *v*_*rel*_. Vertical and horizontal axes represent ***v***_*target*_ and ***v***_*others*_, the group-wise importance scores for the target subtype samples group and the other samples group, respectively.
(EPS)

S3 Fig Intersections of top 500 gene sets calculated by importance analysis of RNA-seq. (a) PWL model and (b) logistic regression model.
(EPS)

S4 Fig Embeddings of deep learning models with RNA-seq features projected by UMAP. The left and right columns are 1D and 2D projected embeddings, respectively. First to fifth rows show embeddings of RV ***η***, RF ***ρ***, SNNs, DeepCC, and DeepCSD, respectively.
(EPS)

S5 Fig PAM50 genes selected as top 500 gene sets in subtypes. Details of PAM50 genes selected for top 500 genes in the deep learning models are shown.
(TIF)

S6 Fig Ideogram of chromosome 17, Cytogenetic. GRCh37ṗ12 (GCF_000001405.24).
(EPS)

S1 Table Intersections of top 500 gene sets of RNA-seq. This table summarizes specific genes (not selected in other subtypes) extracted by PWL model and logistic regression model with RNA-seq features.
(DOCX)

S2 Table Chromosomal location for common genes as specific features of Her2-enriched class in the logistic regression and PWL models. (DOCX)

S1 Appendix Details of PWL model. This appendix summarizes why conventional deep learning models are not explainable and then presents the detail of the PWL model, our explainable deep learning model.
(PDF)

S1 File 10-fold DCV results. This file stores the training and test AUC values of 10-fold DCV results yielded from evaluation with each technique.
(XLSX)

S2 File Hyperparameter optimization results. This file stores the hyperparameter search ranges and their optimized values of each prediction model.
(XLSX)

S3 File Relative scores. This compressed file stores the relative scores of the PWL and logistic regression models.
(ZIP)

S4 File Enriched pathways of deep learning models. This file stores the full list of Table 5 for the copy number features and RNA-seq features.
(XLSX)
