# Supplementary figures and images for: Deep learning generates custom-made logistic regression models for explaining how breast cancer subtypes are classified
Source: PLoS One. 2023 May 22;18(5):e0286072. doi: 10.1371/journal.pone.0286072 (PMC10202302; doi:10.1371/journal.pone.0286072)

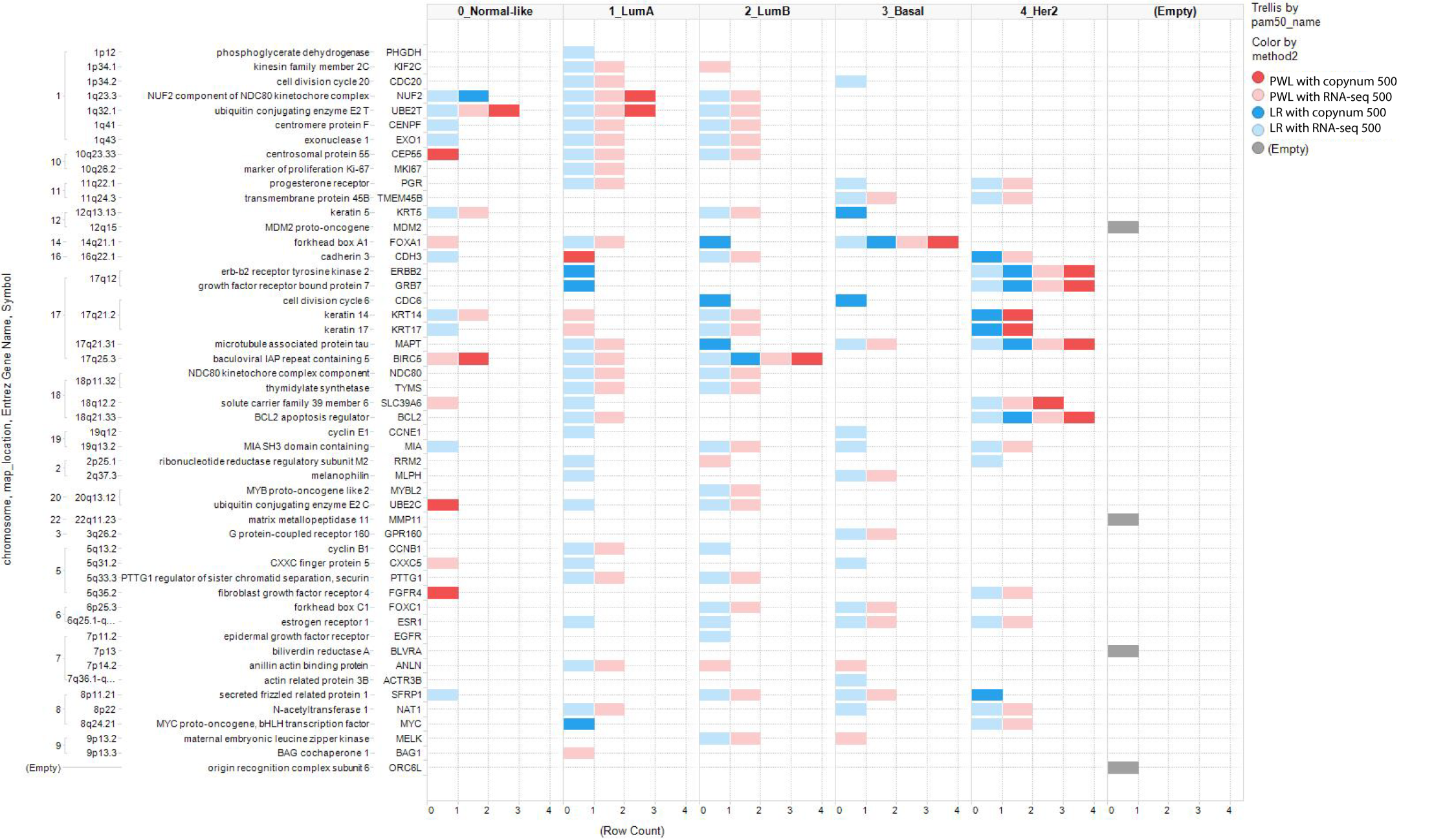

Supplement: S5 Fig — Details of PAM50 genes selected for top 500 genes in the deep learning models are shown. (TIF) [file pone.0286072.s005.tif]
