# Supplementary material for: Deep learning generates custom-made logistic regression models for explaining how breast cancer subtypes are classified
Source: PLoS One. 2023 May 22;18(5):e0286072. doi: 10.1371/journal.pone.0286072 (PMC10202302; doi:10.1371/journal.pone.0286072)
Supplement: S1 Table — This table summarizes specific genes (not selected in other subtypes) extracted by PWL model and logistic regression model with RNA-seq features. (DOCX) [file pone.0286072.s007.docx]

**S1 Table.** Intersections of top 500 gene sets of RNA-seq.

|  |  | Point-wise linear | | | | |
| --- | --- | --- | --- | --- | --- | --- |
|  |  | Normal-like | Luminal A | Luminal B | Basal-like | Her2-enriched |
| Logistic regression | Normal-like | 102 | 0 | 4 | 3 | 1 |
|  | Luminal A | 2 | 64 | 7 | 3 | 1 |
|  | Luminal B | 3 | 5 | 75 | 7 | 3 |
|  | Basal-like | 2 | 7 | 2 | 112 | 4 |
|  | Her2-enriched | 3 | 4 | 1 |  | 165 |
