# Supplementary material for: Deep learning generates custom-made logistic regression models for explaining how breast cancer subtypes are classified
Source: PLoS One. 2023 May 22;18(5):e0286072. doi: 10.1371/journal.pone.0286072 (PMC10202302; doi:10.1371/journal.pone.0286072)
Supplement: S2 Table — (DOCX) [file pone.0286072.s008.docx]

**S2 Table.** Chromosomal location for common genes as specific features of Her2-enriched class in logistic regression and point-wise linear models.

| chr | arm | band | Number of genes |  |
| --- | --- | --- | --- | --- |
| chr10 | q | 11 | 2 |  |
| chr10 | q | 21 | 2 |  |
| chr10 | q | 22 | 3 |  |
| chr17 | q | 11 | 7 |  |
| chr17 | q | 12 | 19 | ERBB2 locus |
| chr17 | q | 21 | 104 |  |
| chr17 | q | 22 | 2 |  |
| chr18 | q | 11 | 10 |  |
| chr18 | q | 12 | 10 |  |
| chr18 | q | 21 | 8 |  |
| chr21 | q | 22 | 7 |  |
| chr4 | p | 12 | 4 |  |
| chr4 | p | 13 | 6 |  |
| chr4 | p | 14 | 3 |  |
| chr4 | q | 21 | 2 |  |
| chr4 | q | 22 | 1 |  |
| chr5 | q | 14 | 3 |  |
| chr8 | p | 11 | 2 |  |
| chr9 | q | 34 | 1 |  |
| unknown |  |  | 10 |  |
| Total |  |  | 206 |  |
