# Supplementary material for: Deep learning generates custom-made logistic regression models for explaining how breast cancer subtypes are classified
Source: PLoS One. 2023 May 22;18(5):e0286072. doi: 10.1371/journal.pone.0286072 (PMC10202302; doi:10.1371/journal.pone.0286072)
Supplement: S1 Appendix — This appendix summarizes why conventional deep learning models are not explainable and then presents the detail of the PWL model, our explainable deep learning model. (PDF) [file pone.0286072.s009.pdf]

S1 Appendix : Point-wise linear models

In this appendix we summarize why usual deep learning models are not explainable and then propose the point-wise linear model, our explainable deep learning model. We assume in this paper that in any vector space the inner product is defined by the dot product. The notation $\boldsymbol{v}(\boldsymbol{u})$ for arbitrary vectors $\boldsymbol{v}$ and $\boldsymbol{u}$ indicates that every element of $\boldsymbol{v}$ is a function of the elements of $\boldsymbol{u}$. Additionally, we use the Einstein summation convention. $a_\alpha b_\alpha \equiv \sum_{\alpha=1}^{D} a_\alpha b_\alpha = a_1 b_1 + a_2 b_2 \cdots a_D b_D$ for arbitrary D-dimensional vectors $\boldsymbol{a}$ and $\boldsymbol{b}$. $A_{\alpha\beta} B_{\beta\gamma} \equiv \sum_{\beta=1}^{D} A_{\alpha\beta} B_{\beta\gamma}$ for arbitrary D-dimensional tensors $A_{\alpha\beta}$ and $B_{\beta\gamma}$. A Greek-character subscript index to indicate components of a vector.

1.1 Idea of point-wise linear models

Let $\boldsymbol{x}^{(n)} \in \mathcal{R}^D$ $(n = 1, \cdots, N)$ represent a feature vector with N denoting the sample size and $\mathcal{R}$ indicating the real number set. Firstly, we defined a logistic regression model as follows:

$$y^{(n)} = \sigma(w_\mu x_\mu^{(n)}), \tag{1.1}$$

where $\boldsymbol{w} \in \mathcal{R}^D$ is a weight vector for $\boldsymbol{x}^{(n)}$, σ is a sigmoid function, and $y^{(n)} := \{y^{(n)} \in \mathcal{R} | 0 \leq y^{(n)} \leq 1\}$ is a probability value. For simplicity of notations in these subsections, bias parameters have been removed from Eq (1.1) and other equations with learning weights. Fig A1 (a) shows the network architecture of the logistic regression model. The weight vector $\boldsymbol{w}$ is bound to the feature vector $\boldsymbol{x}^{(n)}$. We can understand the importance of each feature variable by analyzing the magnitude of the element in $\boldsymbol{w}$.

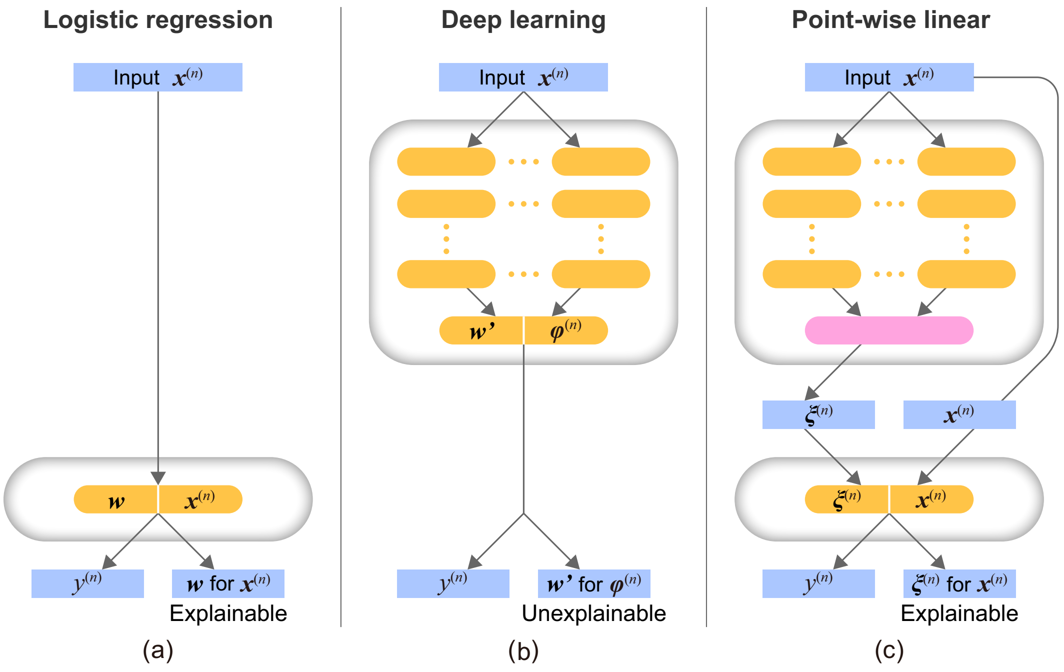

Figure A1: Comparison of network architectures. (a) shows a logistic regression model. $\boldsymbol{x}^{(n)}$ and $y^{(n)}$ are a feature vector and a target value ((n) is sample index), respectively. $\boldsymbol{w}$ is a vector of learning parameters for $\boldsymbol{x}^{(n)}$. (b) shows a fully connected neural network. $\boldsymbol{\varphi}^{(n)}$ and $\boldsymbol{w}'$ are an inner vector and learning parameters, respectively. (c) shows a point-wise linear model. The upper block in (c) is a meta-machine generating a learning parameter $\boldsymbol{\xi}(\boldsymbol{x}^{(n)})$ in Eq (1.5). The lower block in (c) is a logistic regression model for each feature vector $\boldsymbol{x}^{(n)}$.

Next, we give a usual deep learning model as shown in Fig A1(b). A new feature vector $\boldsymbol{\varphi}(\boldsymbol{x}^{(n)}) \in \mathcal{R}^{D'}$ is nonlinearly generated from the original feature vector $\boldsymbol{x}^{(n)}$ through the following L-layer neural network (NN):

$$\boldsymbol{\varphi}(\boldsymbol{x}^{(n)}) = f^{(L)}g^{(L)} \cdots f^{(l)}g^{(l)} \cdots f^{(2)}g^{(2)}f^{(1)}g^{(1)}(\boldsymbol{x}^{(n)}), \tag{1.2}$$

where $f^{(l)}$ is an activation function such as sigmoid, tanh, or ReLU. The function $g^{(l)}$ is expressed as

$$g^{(l)}(\boldsymbol{x}) = \boldsymbol{W}^{(l)}\boldsymbol{x}, \tag{1.3}$$

where $\boldsymbol{W}^{(l)} \in \mathcal{R}^{D^{(l+1)} \times D^{(l)}}$ ($D^{(1)} = D$ and $D^{(L+1)} = D'$) is a weight matrix. The pair of functions $f^{(l)}$ and $g^{(l)}$ is called "the l-th inner layer". A deep-learning-based nonlinear classification function predicts probability $y^{(n)}$ as follows:

$$y^{(n)} = \sigma\left(w'_{\mu}\varphi_{\mu}(\boldsymbol{x}^{(n)})\right), \tag{1.4}$$

where $\boldsymbol{w}' \in \mathcal{R}^{D'}$ is a universal weight vector for $\boldsymbol{\varphi}$. The magnitude of each $\boldsymbol{w}'$ element represents the contribution of the corresponding element of $\boldsymbol{\varphi}$ to the prediction as shown in Fig A1 (b). However, one cannot "explain" the machine's prediction by $\boldsymbol{w}'$ because one cannot understand the meanings of the new feature vector $\boldsymbol{\varphi}$ with which the machine makes its predictions.

In order to make a deep NN explainable, we considered a meta-learning to generate a logistic regression model defined as

$$y^{(n)} = \sigma\left(\xi_{\mu}(\boldsymbol{x}^{(n)})x_{\mu}^{(n)}\right), \tag{1.5}$$

where each element of $\boldsymbol{\xi} \in \mathcal{R}^{D}$ is a function of $\boldsymbol{x}^{(n)}$ that the NN determines. $\boldsymbol{\xi}$ behaves as the weight vector for the original feature vector $\boldsymbol{x}^{(n)}$. The magnitude of each element of $\boldsymbol{\xi}$ describes the importance of the corresponding feature variable. Unlike $\boldsymbol{\varphi}$, the original vector $\boldsymbol{x}^{(n)}$ is comprehensible, and hence the model defined by Eq (1.5) is "explainable." Here we should notice that this weight vector is tailored to each sample because $\boldsymbol{\xi}$ depends on $\boldsymbol{x}^{(n)}$. We call Eq (1.5) a point-wise linear model over the sample index (n). The architecture of the point-wise linear model consists of two blocks as shown in Fig A1(c). The upper block is a meta-learning machine of logistic regression models. The lower block is the logistic regression model for the inference task. In the following subsections we introduce two techniques for constructing the point-wise linear models.

1.2 Straightforward model

As one simple way to construct the weight vector, we considered a matrix $\tilde{\boldsymbol{W}} \in \mathcal{R}^{D \times D'}$ that transforms the nonlinear feature vector $\boldsymbol{\varphi} \in \mathcal{R}^{D'}$ into a vector in the original feature space $\mathcal{R}^{D}$:

$$\eta_{\mu}(\boldsymbol{x}^{(n)}) = \tilde{W}_{\mu\nu}\varphi_{\nu}(\boldsymbol{x}^{(n)}), \tag{1.6}$$

where $\boldsymbol{\eta} \in \mathcal{R}^{D}$. We can construct a point-wise function in a straightforward manner by defining $\boldsymbol{\xi}$ in Eq (1.5) as

$$\boldsymbol{\xi}(\boldsymbol{x}^{(n)}) \equiv \boldsymbol{\eta}(\boldsymbol{x}^{(n)}). \tag{1.7}$$

To train the meta-learning machine Eq (1.5) path through Eq (1.7), we minimized the negative log likelihood (NLL) loss of the output $y^{(n)}$ for the target label $t^{(n)} := \{0, 1\}$:

$$\min \sum_{n=1}^{N} t^{(n)} \log \left[\sigma\left(\xi_\mu(\boldsymbol{x}^{(n)}) x_\mu^{(n)}\right)\right] + (1 - t^{(n)}) \log \left[1 - \sigma\left(\xi_\mu(\boldsymbol{x}^{(n)}) x_\mu^{(n)}\right)\right]. \tag{1.8}$$

To investigate the performance of the explainable point-wise linear model given by Eq (1.5) with (1.7), we trained a simple toy dataset (*sklearn.datasets.make_circle*) by using the straightforward model, the logistic regression model (Eq (1.1)), and a self-normalizing neural networks (SNNs) [1] model as a state-of-the-art NN model. The circle in a circle is not a linearly separable problem, so as shown in Fig A2 (b), the logistic regression model could not classify the two circles. The SNNs correctly classified the blue and orange dots as shown in Fig A2 (c). On the other hand, the point-wise linear model (Eq (1.5) with Eq (1.7)) tried to learn the labels of all samples as it generated a weight vector optimized for each sample, which resulted in failure in Fig A2 (d). The next section describes another approach to generating a point-wise linear function, an approach that improve the generalization ability.

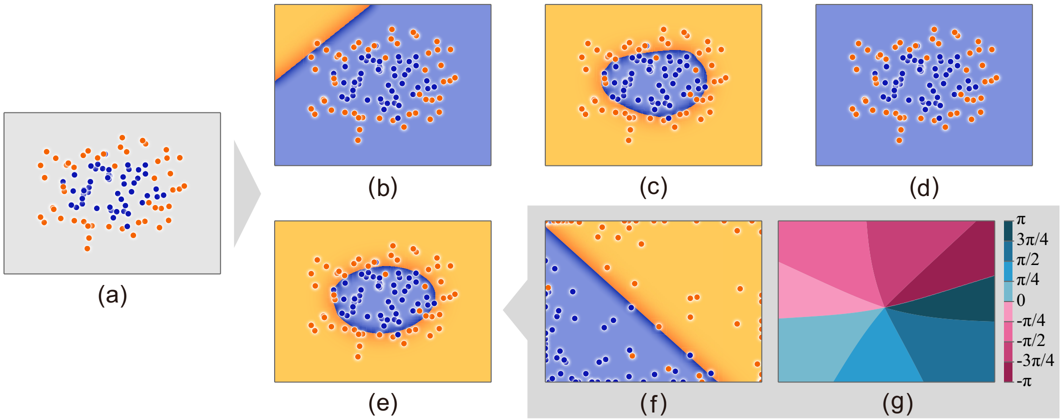

Figure A2: Classification results for a simple toy dataset. (a) is a large circle (orange dots) that contains a smaller circle (blue dots) obtained by *sklearn.datasets.make_circle*. (b) and (c) are the boundaries classified by the logistic regression model and self-normalizing networks (SNNs) model, respectively. (d) and (e) are the boundaries classified by the point-wise linear model of the straightforward model Eq (1.7) and the reallocation-based model Eq (1.9), respectively. (f) is the boundary classified by the reallocated feature vectors $\boldsymbol{\rho}$. (g) is the arctangent of the angle between the horizontal and vertical elements of the weight vector $\boldsymbol{\xi}^{(n)}$. The weight vector smoothly changes for each data sample.

1.3 Reallocation technique

We propose an alternative point-wise linear model by using instead of Eq (1.7)

$$\boldsymbol{\xi}(\boldsymbol{x}^{(n)}) \equiv \boldsymbol{w} \odot \boldsymbol{\eta}(\boldsymbol{x}^{(n)}), \tag{1.9}$$

where $\boldsymbol{\eta}$ is defined in Eq (1.6), $\boldsymbol{w} \in \mathcal{R}^D$ is a universal weight vector that is independent of $\boldsymbol{x}^{(n)}$, $\odot$ is the Hadamard product. $\boldsymbol{\xi}^{(n)} \equiv \boldsymbol{\xi}(\boldsymbol{x}^{(n)})$ gives a weight vector for each sample $\boldsymbol{x}^{(n)}$. As in Eq (1.5), we can analyze the magnitude of $\boldsymbol{\xi}^{(n)}$ for each dimension in the original feature space with which the machine makes decisions. In contrast to the model defined by Eq (1.7), the model defined by Eq (1.9) accurately predicts the classification boundary, as shown in Fig A2(e). The weight vectors $\boldsymbol{\xi}^{(n)}$ in Eq (1.9) smoothly change for each data sample (Fig A2(g)). The reallocation-based point-wise linear model thus enables generalization.

To clarify the mechanism of the alternative point-wise linear model, we reformulated Eq (1.5) with Eq (1.9) as follows:

$$
\begin{aligned}
y^{(n)} &= \sigma\left[\left(\boldsymbol{w}\odot\boldsymbol{\eta}(\boldsymbol{x}^{(n)})\right)_{\mu} x_{\mu}^{(n)}\right] \\
&= \sigma\left[w_{\mu}\left(\boldsymbol{\eta}(\boldsymbol{x}^{(n)})\odot\boldsymbol{x}^{(n)}\right)_{\mu}\right] \\
&\equiv \sigma\left(w_{\mu}\rho_{\mu}(\boldsymbol{x}^{(n)})\right).
\end{aligned}
\tag{1.10}
$$

We call $\boldsymbol{\rho}(\boldsymbol{x}^{(n)}) \equiv \boldsymbol{\eta}(\boldsymbol{x}^{(n)}) \odot \boldsymbol{x}^{(n)}$ a reallocated feature vector in $\mathcal{R}^d$. NNs have a versatile ability to map a linear feature space to a nonlinear feature space. By utilizing this ability, Eq (1.10) reallocates the feature vector $\boldsymbol{x}^{(n)}$ into the new vector $\boldsymbol{\rho}$ that is linearly separable by a single hyperplane drawn by $\boldsymbol{w}$. Additionally, Eq (1.10) can impose a limit on the reallocation by

$$
\boldsymbol{\rho}(\boldsymbol{x}^{(n)}) = C\left(\boldsymbol{\eta}(\boldsymbol{x}^{(n)})\odot\boldsymbol{x}^{(n)}\right), \tag{1.11}
$$

where C is a clamp function that operates on each vector element as follows:

$$
C(x) = \begin{cases} min & \text{if } x < min \\ x & \text{if } min \leq x \leq max \\ max & \text{if } max < x \end{cases}. \tag{1.12}
$$

Fig A2 (f) shows the reallocated feature vectors $\boldsymbol{\rho}(\boldsymbol{x}^{(n)})$; the blue and orange dots are linearly classified. Note that we used C ($min = 0.0$ and $max = 1.0$) in Eq (1.11). As long as the reallocated vector $\boldsymbol{\rho}$ is defined by the Hadamard product operator as in Eq (1.11), one can obtain the weight $\boldsymbol{\xi}^{(n)}$ in the same manner as Eq (1.10).

A framework of multiplicative interactions [2] provides another viewpoint of the point-wise linear model. Eq (1.10) can be reformulated as a special form of multiplicative interaction function between $\boldsymbol{\eta}(\boldsymbol{x}^{(n)})$ and $\boldsymbol{x}^{(n)}$:

$$
y^{(n)} = \sigma(\eta_{\mu}(\boldsymbol{x}^{(n)})W_{\mu\nu}x_{\nu}^{(n)}), \tag{1.13}
$$

where $W_{\mu\nu}$ is restricted as

$$
W_{\mu\nu} = \begin{cases} w_{\mu} & \mu = \nu \\ 0 & \mu \neq \nu \end{cases}. \tag{1.14}
$$

Namely, the reallocation-based point-wise linear can be listed as the variation of self-multiplicative interactions. Various NNs, including RNNs and CNNs, can be used to compose the reallocation vector $\boldsymbol{\eta}$. Moreover, the lower block of the point-wise linear (Fig. A1 (c)) enables all these NNs to explain the machine's prediction with respect to the low-dimensional original feature vector $\boldsymbol{x}$, while that of the upper block can improve the predictions accuracy by using the high-dimensional feature vector $\boldsymbol{\varphi}$.

Regularization

To avoid the overfitting problem, the loss functions of prediction models have the Lp-norm of the learning parameters as the regularization term. Logistic regression models generally use $L1$ and $L2$-norm regularization, which are equivalent to lasso and ridge regression models, respectively [3]. From the viewpoint of the point-wise linear model, the learning parameter is $\boldsymbol{\xi}$, and the regularization terms should correspond to the L1-norm $|\boldsymbol{\xi}|_{L1}$ and L2-norm $|\boldsymbol{\xi}|_{L2}$. The Lp ($p = 1, 2$) regularization term is given by

Table A1: Variations of the reallocation functions.

Type	Transform function	Transform weight	Reallocation function
I	$\boldsymbol{u}^{(n)} = \boldsymbol{\eta}(\boldsymbol{x}^{(n)})$	$\tilde{W} \in \mathcal{R}^{d \times d^{(L)}}$	$\boldsymbol{\rho}(\boldsymbol{x}^{(n)}) = \boldsymbol{u}^{(n)} \odot \boldsymbol{x}^{(n)}$
II	$\boldsymbol{u}^{(n)} = \boldsymbol{\eta}(\boldsymbol{x}^{(n)})$	$\tilde{W} \in \mathcal{R}^{d \times d^{(L)}}$	$\boldsymbol{\rho}(\boldsymbol{x}^{(n)}) = \boldsymbol{u}^{(n)} + \boldsymbol{x}^{(n)}$
III	$(\boldsymbol{u}^{(n)}, \boldsymbol{v}^{(n)}) = \boldsymbol{\eta}(\boldsymbol{x}^{(n)})$	$\tilde{W} \in \mathcal{R}^{2d \times d^{(L)}}$	$\boldsymbol{\rho}(\boldsymbol{x}^{(n)}) = \boldsymbol{u}^{(n)} \odot (\boldsymbol{x}^{(n)} + \boldsymbol{v}^{(n)})$
IV	$(\boldsymbol{u}^{(n)}, \boldsymbol{v}^{(n)}) = \boldsymbol{\eta}(\boldsymbol{x}^{(n)})$	$\tilde{W} \in \mathcal{R}^{2d \times d^{(L)}}$	$\boldsymbol{\rho}(\boldsymbol{x}^{(n)}) = (\boldsymbol{u}^{(n)} \odot \boldsymbol{x}^{(n)}) + \boldsymbol{v}^{(n)}$

$$Lp\left(\boldsymbol{\xi}(\boldsymbol{x}^{(n)})\right) = \left|\boldsymbol{w} \odot \boldsymbol{\eta}(\boldsymbol{x}^{(n)})\right|_p . \tag{1.15}$$

The above equation constrains the range (magnitude) of $\boldsymbol{\eta}(\boldsymbol{x}^{(n)})$. The Lp-norm reduces the cost of the reallocation of the original feature vector $\boldsymbol{x}^{(n)}$. It induces sparseness in the network pathway of $\boldsymbol{\varphi}$. Additionally, Eq (1.15) can be extended to an elastic-net regularization term as follows:

$$L_{elastic}\left(\boldsymbol{\xi}(\boldsymbol{x}^{(n)})\right) = \alpha L_1\left(\boldsymbol{\xi}(\boldsymbol{x}^{(n)})\right) + (1-\alpha) L_2\left(\boldsymbol{\xi}(\boldsymbol{x}^{(n)})\right), \tag{1.16}$$

where α is a mixing parameter with $0 \leq \alpha \leq 1$.

Variants

The necessary condition for constructing a point-wise function is that the reallocation function is linear (see Table A1). These reallocation functions allow us to calculate an element-wise magnitude of the point-wise learning weight for each feature. For example, the learning weight and bias term of the type II (adaptive reallocation) function are $w_\mu u_\mu^{(n)}$ and $w_\mu x_\mu^{(n)}$, respectively. The functions of types I-IV have almost equal prediction abilities, as shown in Fig A3. Each panel shows the classification boundaries in feature vectors (generated by *sklearn.datasets.make_moon*). The type I (multiplicative reallocation) vector $\boldsymbol{\rho}$ is equivalent to the weight of the logistic regression-type linear models, so medical researchers familiar with logistic regression may find the properties of type I especially useful.

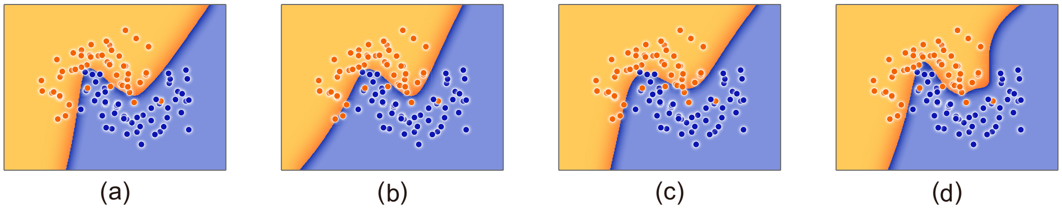

Figure A3: Classification results for each reallocation function. The dataset is *sklearn.datasets.make_moon*. Each panel shows the classification boundary obtained by one of the following the reallocation functions: (a) $\boldsymbol{u}^{(n)} \odot \boldsymbol{x}^{(n)}$, (b) $\boldsymbol{u}^{(n)} + \boldsymbol{x}^{(n)}$, (c) $\boldsymbol{u}^{(n)} \odot (\boldsymbol{x}^{(n)} + \boldsymbol{v}^{(n)})$, or (d) $(\boldsymbol{u}^{(n)} \odot \boldsymbol{x}^{(n)}) + \boldsymbol{v}^{(n)}$.

Unified architecture

NNs (Fig A1(b)) likely cause overfitting when the number of layers L is increased [3]. A possible reason for this phenomenon is that the progression of learning is confined to the upper-layer parameters; i.e., there is a lack of advanced learning in the lower layers. Residual NNs can be used to tackle this problem by adding skip connections [4, 5]. This approach mixes the current and previous inner-layer outputs and transfers the mixed output to the next layer. The lower inner layers can get gradients during the back-propagation process. We used this skip-connection approach and developed

an architecture characterized by the neurons being bound to each network layer in a mesh-like form as shown in Fig A4. We named this architecture deep unified networks (DUNs) [6]. The mesh connections provide gradients to all layers to train their weights. Here we propose a black box breaker (B3) consisting of compound NNs of the DUNs and the point-wise linear function. In B3, DUNs construct a vector that corresponds to $\boldsymbol{\eta}$ in Eq (1.6) for each inner layer. As a result, B3 explains the inner layers' decisions in the original feature space.

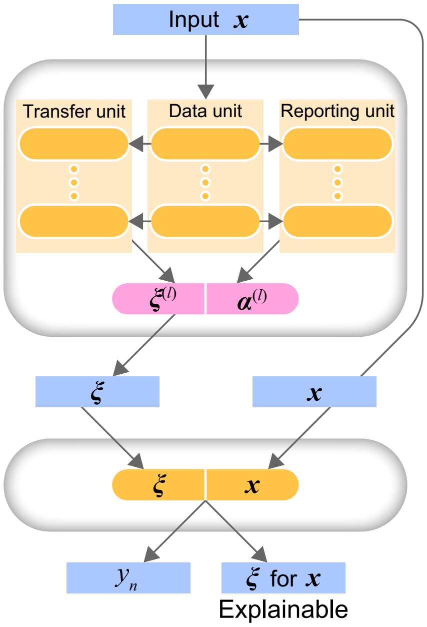

Figure A4: Network architecture of black box breaker (B3). Deep unified networks (DUNs) outputs the weight of the logistic regression for each layer and sample.

In this subsection, we omit the sample index (n) for simplicity. B3 consists of data units, transform units, reporting units, an attention unit, and a classification unit as shown in Fig A4. The data units correspond to the inner layers of conventional NNs. The data units are defined as follows:

$$\boldsymbol{z}^{(l+1)} = f^{(l)} g^{(l)}(\boldsymbol{z}^{(l)}), \tag{1.17}$$

where $\boldsymbol{z}^{(l)} \in \mathcal{R}^{D^{(l)}}$ $(\boldsymbol{z}^{(1)} = \boldsymbol{x})$ represents the input and output of the data units. $f_{\mu}^{(l)}$ is an activation function such as sigmoid, tanh, or ReLU. $g^{(l)}$ is a function with a learning weight such as Eq (1.3). When there are more than two layers, we use highway networks [5] as $g^{(l)}$.

The transform units convert $\boldsymbol{z}^{(l)} \in \mathcal{R}^{D'}$ into a vector $\boldsymbol{\eta}^{(l)}$ in the original feature space $\mathcal{R}^{d}$:

$$\boldsymbol{\eta}^{(l)}(\boldsymbol{z}^{(l)}) = \tilde{\boldsymbol{W}}^{(l)} \boldsymbol{z}^{(l)}, \tag{1.18}$$

where $\tilde{\boldsymbol{W}}^{(l)} \in \mathcal{R}^{D \times D^{(l)}}$ is a weight matrix. The transform units are bound to the data units when $l \geq 2$.

To extract the abstract information of the data unit vector $\boldsymbol{z}^{(l)}$, the reporting units compress the $D^{(l)}$-dimensional vector $\boldsymbol{z}^{(l)}$ into a D_R-dimensional vector $\boldsymbol{r}^{(l)}$. The reporting units are defined as follows:

$$\boldsymbol{r}^{(l)}(\boldsymbol{z}^{(l)}) = \sigma(\hat{\boldsymbol{W}}^{(l)} \boldsymbol{z}^{(l)}), \tag{1.19}$$

where $\hat{\boldsymbol{W}}^{(l)} \in \mathcal{R}^{D_R \times D^{(l)}}$ is a weight matrix, and σ is a sigmoid function applied to each element of the vector. The reporting units are bound to the data units when $l \geq 2$.

From $\boldsymbol{r}^{(l)}$ an attention unit calculates attention weights for each layer. In particular, we use a dot-attention unit that calculates the attention weights $a^{(l)}$ as

$$a^{(l)} = \mathrm{softmax}\left(\tilde{\boldsymbol{w}}\left(\frac{\boldsymbol{R}\boldsymbol{R}^{\top}}{\sqrt{L}}\right)\right)^{(l)}, \tag{1.20}$$

where $\tilde{\boldsymbol{w}} \in \mathcal{R}^{L}$ is a learnable key vector for the attention and the softmax function serves as an activation function. The matrix $\boldsymbol{R} \in \mathcal{R}^{L \times D_R}$ is composed by stacking the reporting vectors $\{\boldsymbol{r}^{(2)}, \cdots, \boldsymbol{r}^{(L+1)}\}$. $\boldsymbol{R}^{\top}$ is the transposed matrix of $\boldsymbol{R}$. A similar attention mechanism was used in Transformer [7]. We used the learnable key $\tilde{\boldsymbol{w}}$ in our classification application.

B3 evaluates the attention averaged transform vector $\bar{\boldsymbol{\eta}}$ as

$$\bar{\boldsymbol{\eta}}(\boldsymbol{x}) = \sum_{l=2}^{L+1} a^{(l)} \boldsymbol{\eta}^{(l)}(\boldsymbol{z}^{(l)}). \tag{1.21}$$

$\bar{\boldsymbol{\eta}}$ is determined by running through the feature pathway (Eqs (1.17) and (1.18)) and the attention pathway (Eqs (1.17), (1.19), and (1.20)). The number of inner layers of the transform function is interpreted as the number of data units L in the feature pathway. This architecture is a fully connected attention network similar to the transposed form of the attention networks of RNNs in [8].

Finally, the point-wise linear model broke the black box of the NNs. In B3 we used the attention averaged transform vector $\bar{\boldsymbol{\eta}}$ instead of $\boldsymbol{\eta}$ in Eq (1.9):

$$y = \sigma\left[\left(\boldsymbol{w} \odot \bar{\boldsymbol{\eta}}(\boldsymbol{x})\right)_{\mu} x_{\mu}\right], \tag{1.22}$$

$$= \sigma\left[\left(\sum_{l=2}^{L+1} a^{(l)} \xi_{\mu}^{(l)}(\boldsymbol{x})\right) x_{\mu}\right], \tag{1.23}$$

$$= \sigma\left(\bar{\xi}_{\mu}(\boldsymbol{x}) x_{\mu}\right), \tag{1.24}$$

where $\boldsymbol{\xi}^{(l)} \equiv \boldsymbol{w} \odot \boldsymbol{\eta}^{(l)}$ and $\bar{\boldsymbol{\xi}} \equiv \boldsymbol{w} \odot \bar{\boldsymbol{\eta}}$. B3 provides the magnitude of $a^{(l)}\boldsymbol{\xi}^{(l)}$ as the feature importance for each layer and sample and is a fully explainable deep architecture. In addition, the input layer is connected directly to the neurons of the output layer as shown in Eq (1.22). The long skip connection of the B3 architecture avoids the overfitting problem.

References

[1] Günter Klambauer, Thomas Unterthiner, Andreas Mayr, and Sepp Hochreiter. Self-normalizing neural networks. *CoRR*, abs/1706.02515, 2017.

[2] Siddhant M Jayakumar, Wojciech M Czarnecki, Jacob Menick, Jonathan Schwarz, Jack Rae, Simon Osindero, Yee Whye Teh, Tim Harley, and Razvan Pascanu. Multiplicative interactions and where to find them. In *International Conference on Learning Representations*, 2019.

[3] Jerome Friedman, Trevor Hastie, and Robert Tibshirani. *The elements of statistical learning*, volume 1. Springer series in statistics New York, NY, USA:, 2001.

[4] Kaiming He, Xiangyu Zhang, Shaoqing Ren, and Jian Sun. Deep residual learning for image recognition. *CoRR*, abs/1512.03385, 2015.

[5] Rupesh Kumar Srivastava, Klaus Greff, and Jürgen Schmidhuber. Highway networks. *CoRR*, abs/1505.00387, 2015.

[6] Sara Bersche Golas, Takuma Shibahara, Stephen Agboola, Hiroko Otaki, Jumpei Sato, Tatsuya Nakae, Toru Hisamitsu, Go Kojima, Jennifer Felsted, Sujay Kakarmath, et al. A machine learning model to predict the risk of 30-day readmissions in patients with heart failure: a retrospective analysis of electronic medical records data. *BMC medical informatics and decision making*, 18(1):44, 2018.

[7] Ashish Vaswani, Noam Shazeer, Niki Parmar, Jakob Uszkoreit, Llion Jones, Aidan N. Gomez, Lukasz Kaiser, and Illia Polosukhin. Attention is all you need. *CoRR*, abs/1706.03762, 2017.

[8] Minh-Thang Luong, Hieu Pham, and Christopher D Manning. Effective approaches to attention-based neural machine translation. *arXiv preprint arXiv:1508.04025*, 2015.
